# MET Is Required for the Maximal Action of 20-Hydroxyecdysone during *Bombyx* Metamorphosis

**DOI:** 10.1371/journal.pone.0053256

**Published:** 2012-12-27

**Authors:** Enen Guo, Qianyu He, Shumin Liu, Ling Tian, Zhentao Sheng, Qin Peng, Jingmin Guan, Mingan Shi, Kang Li, Lawrence I. Gilbert, Jian Wang, Yang Cao, Sheng Li

**Affiliations:** 1 From Key Laboratory of Insect Developmental and Evolutionary Biology, Institute of Plant Physiology and Ecology, Shanghai Institutes for Biological Sciences, Chinese Academy of Sciences, Shanghai, China; 2 Laboratory of Insect Molecular Biology and Biotechnology, Guangdong Provincial Key Laboratory of Agro-animal Genomics and Molecular Breeding, College of Animal Sciences, South China Agricultural University, Guangzhou, China; 3 Department of Biology, University of North Carolina, Chapel Hill, North Carolina, United States of America; 4 Department of Entomology, University of Maryland, College Park, Maryland, United States of America; University of Geneva, Switzerland

## Abstract

Little is known about how the putative juvenile hormone (JH) receptor, the bHLH-PAS transcription factor MET, is involved in 20-hydroxyecdysone (20E; the molting hormone) action. Here we report that two MET proteins found in the silkworm, *Bombyx mori*, participate in 20E signal transduction. *Met* is 20E responsive and its expression peaks during molting and pupation, when the 20E titer is high. As found with results from RNAi knockdown of *EcR*-*USP* (the ecdysone receptor genes), RNAi knockdown of *Met* at the early wandering stage disrupts the 20E-triggered transcriptional cascade, preventing tissue remodeling (including autophagy, apoptosis and destruction of larval tissues and generation of adult structures) and causing lethality during the larval-pupal transition. MET physically interacts with EcR-USP. Moreover, MET, EcR-USP and the 20E-response element (EcRE) form a protein-DNA complex, implying that MET might modulate 20E-induced gene transcription by interacting with EcR-USP. In conclusion, the 20E induction of MET is required for the maximal action of 20E during *Bombyx* metamorphosis.

## Introduction

The molting hormone, 20-hydroxyecdysone (20E), and juvenile hormone (JH) coordinately control insect molting and metamorphosis. Overall, 20E orchestrates the molting process, whereas JH determines the nature of the molt. In the fruitfly, *Drosophila melanogaster*, Methoprene-tolerant (MET), a bHLH-PAS transcription factor [1], binds JH at physiological concentrations *in vitro* [2] and is postulated to be the JH receptor [3]. MET forms homodimers or heterodimers with its paralog, germ-cell expressed (GCE), and JH reduces this dimerization [4]. Although *Met* and *gce* null single mutants are fully viable, *Met gce* double mutants die during the larval-pupal transition [5], [6], resembling what is seen in JH-deficient animals [7]. Functionally, MET/GCE mediates JH action to prevent 20E-triggered apoptosis of larval fat body [6], [7] and differentiation of the optic lobe of the adult brain [8]. In the beetle *Tribolium castaneum*, MET plays a similar key role in JH action during the larval-pupal metamorphosis [9], [10]. Recently, the ligand binding properties of MET were confirmed in *Tribolium*, suggesting strongly that MET is the actual JH receptor [11].

A great deal more is known about the 20E signal transduction pathway. The 20E nuclear receptor complex is a heterodimer composed of ecdysone receptor (EcR) and ultraspiracle (USP) [12], [13]. The heterodimeric EcR-USP is known as the ecdysone receptor and binds the 20E-response element (EcRE) with the assistance of a molecular chaperone complex [14]. In the absence of 20E, the ecdysone receptor associates with transcriptional co-repressors. When 20E binds to the ecdysone receptor, the co-repressors dissociate [15], [16]. The ligand-receptor complex (20E-ecdysone receptor complex) then recruits transcriptional co-activators to induce gene expression through the EcRE [17]. 20E triggers a transcriptional cascade, including transcription of the 20E primary-response genes (i.e. transcription factor genes *Br-C*, *E74*, *E75*, and *E93*) and, subsequently, the 20E secondary-response genes [18]. Moreover, *Br-C*, *E74*, *E75*, *E93* and other 20E response genes positively impact 20E signaling. For example, E93 binds to many 20E response genes and cell death genes on polytene chromosomes. The expression of these genes is defective in *E93* mutants, while *E93* overexpression results in the upregulation of these genes [19].

One major function of JH is to inhibit some of the actions of 20E [3]. The p160/SRC/NCoA-like molecule, TAIMAN in *Drosophila* [20] and FISC in the mosquito, *Aedes aegypti* [21], which also belongs to the bHLH-PAS family of transcriptional regulators, is a transcriptional co-activator of the 20E-ecdysone receptor complex through physical interaction with EcR. Moreover, the p160/SRC/NCoA-like molecule directly associates with MET and is involved in JH action in *Aedes*, *Drosophila*, and *Tribolium*, suggesting a role in enhancing JH-20E crosstalk [22], [23]. It has been shown that the orphan nuclear receptor βFTZ-F1, which is a competence factor for the 20E-ecdysone receptor complex [24], is also involved in JH action [25], [26].

Previously, we performed RNAi knockdown studies of the ecdysone receptor (*EcR*-*USP* RNAi) during the early wandering stage in the silkworm, *Bombyx mori*. *EcR-USP* RNAi was shown to disrupt the 20E-triggered transcriptional cascade, preventing tissue remodeling and resulting in lethality during metamorphosis [27], [28], [29]. Surprisingly, RNAi knockdown of *Met* (*Met* RNAi) during this stage resembles the data resulting from *EcR-USP* RNAi. MET physically interacts with EcR-USP, which forms a protein-DNA complex with the 20E-response element (EcRE) supporting the conclusion that MET is required for the maximal action of 20E during metamorphosis in *Bombyx*.

## Results

### The two *Met* genes are 20E responsive

**Figure 1:**
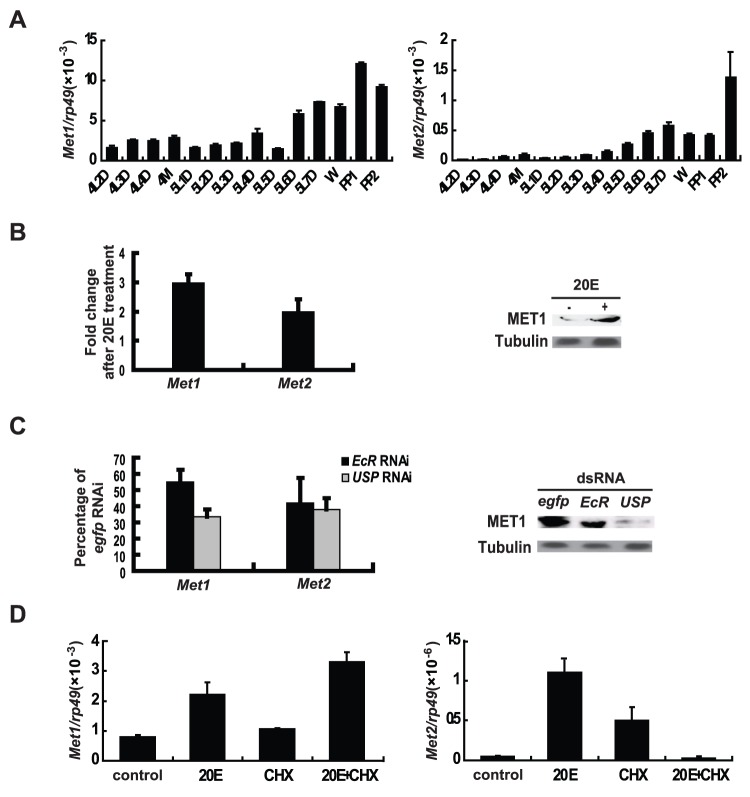
Two *Met* genes in the *Bombyx* genome. Three biological replicates were used, one of which is represented. In each biological replicate, more than 10 larvae were used (A–C). (A) From day 2 of the 4^th^ instar to day 2 of the prepupal stage, *Met1* and *Met2* mRNA expression in fat body was determined by qPCR. The developmental profiles show expression peaks during molting and pupation. 4L2D, day 2 of the fourth instar, and so on; M, molting; W, the wandering stage; PP, the prepupal stage. (B) *Met1* and *Met2* mRNA levels (left panel), and MET1 protein level (right panel) were increased by 20E treatment *in vivo*. 20E (1 µg per larva) was injected into selected larvae on day 2 of the fifth instar, and fat body was explanted for qPCR analysis and Western blots 6 hr after 20E treatment. Tubulin was used as a loading control. (C) *Met1* and *Met2* mRNA levels (left panel), and MET1 protein level (right panel) were decreased by *EcR* RNAi and *USP* RNAi *in vivo*. dsRNA (10 µg per larva) was injected into larvae during the initiation of the early wandering stage, and fat body was explanted for qPCR analysis and Western blots 24 hr after RNAi treatment. Tubulin was used as a loading control. (D) Simultaneous addition of 1 µM 20E and 10 µg/ml cycloheximide (CHX) to *Bombyx* DZNU-Bm-12 cells for 2 hr revealed that *Met1* and *Met2* are 20E primary- and secondary-response genes, respectively.

There are two *Met* genes, *Met1* and *Met2*, in the *Bombyx* genome (GenBank accession numbers: *Met1*, EU249371; *Met2*, EU249372) (Figure S1A) [30]. *Met1* and *Met2* mRNA expression in the fat body was measured from day 2 of the 4^th^ instar to day 2 of the prepupal stage by quantitative real-time PCR (qPCR). The developmental profiles show that *Met* mRNA levels reach a small peak during the 4^th^ larval molt and are very high during the larval-pupal transition (Figure 1A), suggesting that they are upregulated at stages when the 20E titer is high [31]. *Met1* and *Met2* mRNA levels as well as the MET1 protein level were increased in the fat body 6 hr after 20E injection into day 2 of the 5^th^ instar larvae (Figure 1B and S1B). They were also decreased 24 hr after *EcR*-*USP* RNAi at the initiation of the early wandering stage (Figure 1C). Furthermore, simultaneous addition of 20E and the protein synthesis inhibitor cycloheximide to the *Bombyx* DZNU-Bm-12 cells revealed that *Met1* and *Met2* were 20E primary- and secondary-response genes, respectively (Figure 1D). In general, the *Met1* mRNA level in the fat body is much higher than the *Met2* mRNA level. These data imply roles for the *Met* genes during metamorphosis. To further substantiate this, premise RNAi studies were conducted.

### *Met* RNAi results in lethality

**Figure 2:**
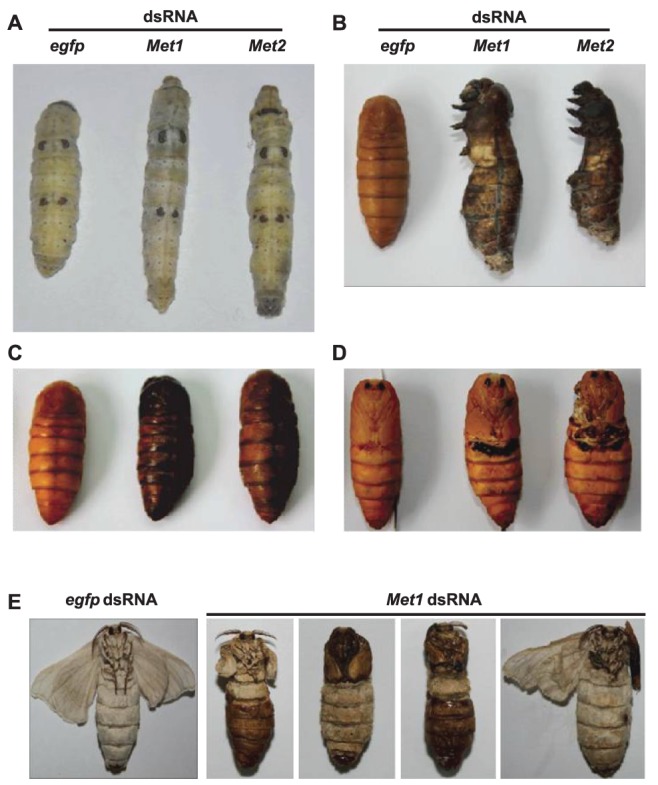
Lethal and defective phenotypes caused by *Met* RNAi in silkworms. dsRNA (10 µg per larva) was injected into selected larva during initiation of the early wandering stage. *egfp* dsRNA was used as a control. (A–C) Typical *Met1* RNAi and *Met2* RNAi treated silkworms died during the wandering stage (A) or during pupation (B), while some were arrested at the mid-pupal stage (C). The pictures (A–C) show the dying animals after *Met* RNAi. (D, E) *Met* RNAi affected adult structure formation. The surviving *Met1* RNAi and *Met2* RNAi treated pupae did not fully develop legs and wings during the late pupal stage (D). Many of the surviving *Met1* RNAi adults failed to shed the pupal cuticle attached to their head or abdomen, exhibiting shortened and distorted legs or unexpanded wings (E).

*Met* RNAi (10 µg dsRNA per larva) was performed at the initiation of the early wandering stage. *Met* RNAi resulted in lethality during the larval-pupal-adult metamorphosis, with a higher percentage of lethality occurring from *Met2* RNAi (∼80%) compared to *Met1* RNAi (∼50%) (Table 1). Although most of the *Met* RNAi treated silkworms were able to spin, their cocoons were much thinner (Figure S2A), and the larval-pupal transition was delayed significantly (∼24 hr) (Figure 2A). Some *Met* RNAi treated silkworms died during the wandering stage (Figure 2A) or during pupation (Figure 2B), while some arrested during the mid-pupal stage lacked adult structures (Figure 2C). Overall, *Met* RNAi results in lethal phenotypes similar to *EcR*-*USP* RNAi treated animals [27], demonstrating that MET is functionally important during *Bombyx* metamorphosis.

**Table 1:** *Met* RNAi results in lethality during the larval-pupal-adult metamorphosis.

dsRNA	Treatment	Injected larvae	Larval lethality,%	Prepupal lethality,%	Pupal lethality,%	Total lethality,%
*egfp*	10ug/larva	87	0	3.5	0	3.5
*Met1*	10ug/larva	92	15.2	13.1	21.7	50
*Met2*	10ug/larva	91	30.7	18.7	29.7	79.1
*Met1+2*	5+5ug/larva	97	8.2	19.6	23.7	52.5

dsRNA (10 µg per larva) was injected into selected larvae during initiation of the early wandering stage. Lethality was scored at the larval, prepupal, and pupal stages to compare the effects of *egfp*, *Met1*, *Met2*, and *Met1*+*2* dsRNAs.

### *Met* RNAi prevents tissue remodeling

Through the ecdysone receptor, the 20E-triggered transcriptional cascade is important in removing obsolete larval tissues via programmed cell death (PCD, mainly apoptosis and autophagy) and generating adult structures from progenitor cells during metamorphosis [18], [32].

**Figure 3:**
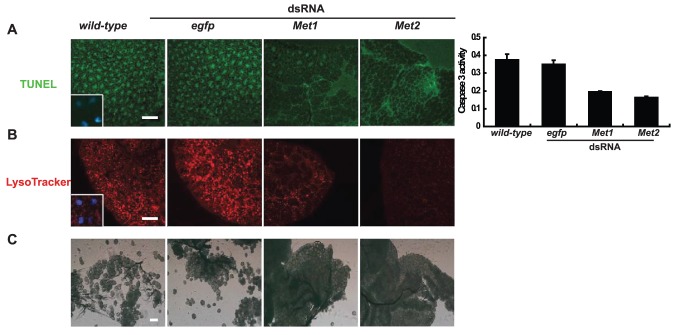
*Met* RNAi prevents fat body remodeling. The inhibitory effects on fat body remodeling by *Met2* RNAi during the initiation of the early wandering stage were stronger than for *Met1* RNAi (A–C). *egfp* dsRNA was used as a control. (A) *Met* RNAi prevented the apoptotic events, estimated by TUNEL (left panel, green) and measured by caspase 3 activity (right panel) 18 hr after RNAi treatment. The inset shows that TUNEL (green) and DAPI (blue) co-localize in nuclei (Bar: 50 µm). (B) *Met* RNAi prevented autophagy, estimated by LysoTracker (red) 24 hr after RNAi treatment. The inset shows that the LysoTracker (red) and DAPI (blue) stain the cytoplasm and the nuclei, respectively (Bar: 50 µm). (C) *Met* RNAi dramatically prevented cell dissociation of the fat body 24 hr after pupation (Bar: 50 µm).

Since *Met* RNAi treated animals results in lethal phenotypes similar to those observed in *EcR*-*USP* RNAi treated animals, we investigated the effects of *Met* RNAi on larval tissue remodeling during *Bombyx* metamorphosis to determine the possible role of MET in PCD. Eighteen hr after treatment, *Met* RNAi significantly prevented apoptotic events in the fat body as estimated by TUNEL labeling and quantification of caspase 3 activity [29] (Figure 3A). By 24 hr, *Met* RNAi nearly abolished autophagy, as estimated by LysoTracker staining [33], [34] (Figure 3B). Twenty-four hr after pupation, *Met* RNAi dramatically inhibited fat body cell dissociation (Figure 3C). The inhibitory effects on fat body tissue remodeling by *Met2* RNAi were stronger than for *Met1* RNAi (Figure 3A–C). Similar to the fat body, silk gland lysis was also prevented by *Met* RNAi 24 hr after pupation. In this tissue, the inhibitory effects of *Met2* RNAi were also stronger than for *Met1* RNAi (Figure S2B).

*Met* RNAi also affected adult structure formation. Most of the surviving *Met* RNAi pupae did not fully develop legs or wings during the late pupal stage (Figure 2D). Since half of the *Met1* RNAi treated larvae survived to the adult stage, we closely examined developmental defects of their adult structures. Many of the surviving *Met1* RNAi adults failed to emerge normally (pupal cuticle remained attached to the head or abdomen) and they had shortened and distorted legs or unexpanded wings (Figure 2E and Figure S2C). These results demonstrate that MET is required for proper tissue remodeling during *Bombyx* metamorphosis, including PCD of obsolete larval tissues and generation of adult structures. We next examined the question of the mechanism of MET action.

### *Met* RNAi disrupts the 20E-triggered transcriptional cascade

**Figure 4:**
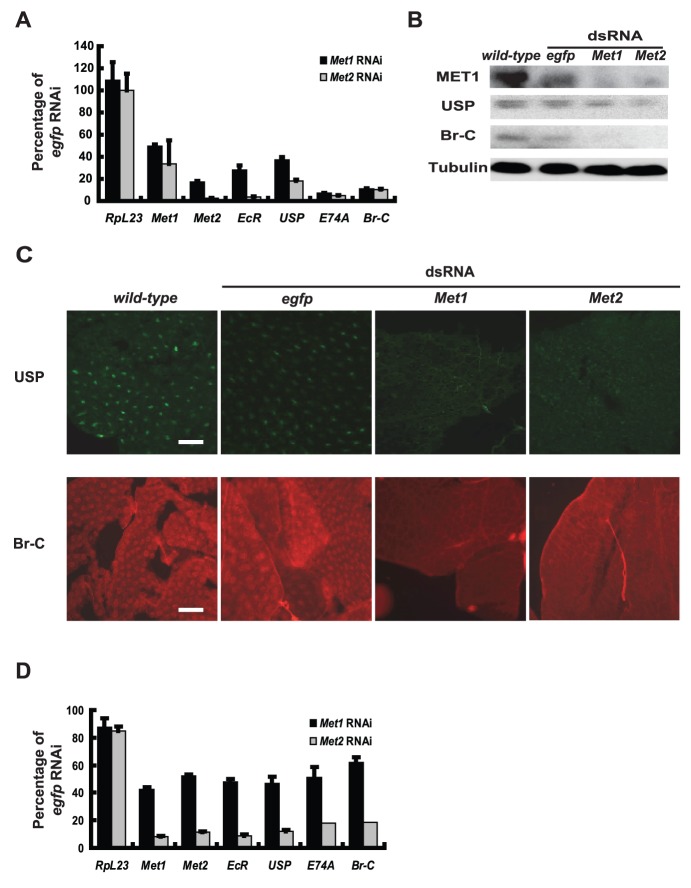
*Met* RNAi disrupts the 20E-triggered transcriptional cascade. RNAi was performed during initiation of the early wandering stage (A–C) and ∼6 hr after pupation (D). The RNAi knockdown efficiency by *Met2* RNAi is higher than for *Met1* RNAi, and the downregulation rate of *Met1* by *Met2* RNAi is higher than for *Met2* by *Met1* RNAi. The #1 set of *Met* dsRNA was used. *egfp* dsRNA was used as a control. (A) *Met1* and *Met2*, and the 20E-response genes *EcR*, *USP*, *E74A* and *Br-C*, as determined by qPCR, were significantly downregulated 24 hr after *Met* RNAi. *RpL23* is used as a negative control of 20E-response gene. (B) MET1, USP and Br-C protein levels, as determined by Western blots, significantly decreased 24 hr after *Met* RNAi. Tubulin was used as a loading control. (C) USP and Br-C protein levels, as estimated by immunohistochemistry, significantly decreased 24 hr after *Met* RNAi. Localization of USP (green) and Br-C (red) were restricted to nuclei (Bar: 50 µm). (D) RNAi was performed ∼6 hr after pupation. The rest is as in (A).

Since the *Met* RNAi effects resemble those of *EcR*-*USP* RNAi at the phenotypic level, we investigated whether *Met* RNAi also disrupts the 20E-triggered transcriptional cascade in the fat body. As determined by qPCR, the 20E-response genes *EcR*, *USP*, *Br-C*, and *E74A* were significantly downregulated 24 hr after *Met* RNAi. Similar to the phenotypic effects of *Met* RNAi, the inhibitory effects on gene expression by *Met2* RNAi (70–95%) were stronger than for *Met1* RNAi (50–90%) (Figure 4A). Moreover, Western blots for MET1, USP, and Br-C (Figure 4B) as well as immunohistochemistry for USP and Br-C (Figure 4C) revealed that protein levels were decreased by *Met* RNAi. To avoid the possibility of off-targeting, we generated two other sets of *Met* dsRNAs (Figure S1A) which exhibited similar but stronger inhibiting effects on gene expression when higher concentrations (30 µg of dsRNA per larva) were used (Figure S3A). Since three different sets of *Met* dsRNAs were used, the off-targeting problem should be largely minimized. To be consistent with the above experimental data (Figures 2, 3, 4 and S2), we still used the first set of *Met* dsRNAs in the following experiments.

To verify the above results, samples of fat body were explanted 24 hr after RNAi treatment and cultured for an additional 6 hr in the presence of 20E. 20E treatment caused significant upregulation of all 6 genes in the *egfp* RNAi treated fat body, while this upregulation was dramatically decreased when *Met2* RNAi was performed (Figure S3B). We therefore conclude that *Met* RNAi disrupts the 20E-triggered transcriptional cascade in the fat body during larval-pupal metamorphosis.

To prevent effects of JH, *Met* RNAi experiments were performed ∼6 hr after pupation, a stage where JH is absent and 20E is present [30]. Surprisingly, both *EcR*-*USP* RNAi and *Met* RNAi at this stage did not cause lethality. However, the 20E-response genes *EcR*, *USP*, *Br-C*, and *E74A* were significantly downregulated 24 hr after *Met* RNAi treatment, and the inhibitory effects of *Met2* RNAi were stronger than for *Met1* RNAi (Figure 4D).

To further insure that the interference was not due to JH, we performed *Met* RNAi followed by the addition of 20E to *Bombyx* DZNU-Bm-12 cells [35] which should lack JH. *Met2* mRNA levels were very low in these cells and the efficiency of *Met2* RNAi was poor, but *Met1* RNAi decreased *Met1* mRNA levels by about 90%. Six hr after 20E treatment, all the 20E-response genes were significantly upregulated in *egfp* RNAi treated cells, but this upregulation was significantly decreased in *Met1* RNAi treated cells (Figure S3C). These data show conclusively that MET is required for the maximal ability of 20E to induce gene expression, and thus tissue remodeling, in *Bombyx*.

In addition, RNAi knockdown of either *EcR* or *Met* in *Tribolium* during the early quiescent stage resulted in lethality, delayed larval-pupal transition, and disrupted the 20E-triggered transcriptional cascade (Figure S4), demonstrating that MET is also required for the maximal action of 20E during metamorphosis in *Tribolium*. We then turned to the question of transactivation of the 20E-ecdysone receptor complex by MET in *Bombyx*.

### MET, EcR-USP and EcRE are components of a protein-DNA complex

**Figure 5:**
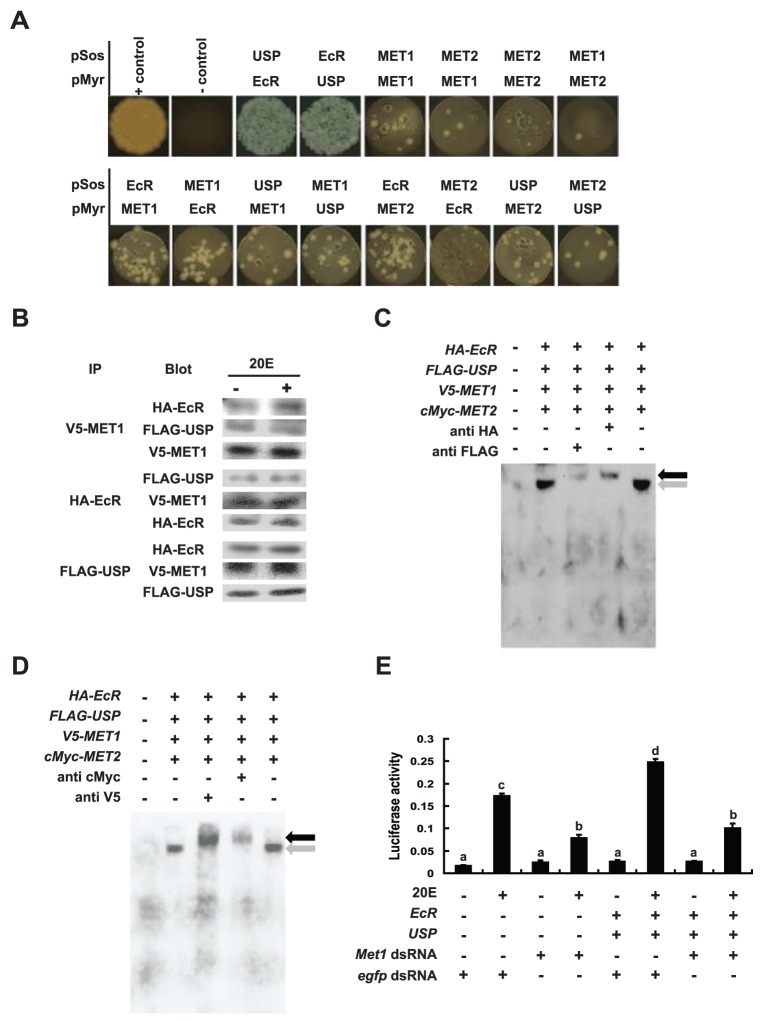
Physical interaction between MET and EcR-USP. (A) The CytoTrap yeast two-hybrid analyses revealed direct associations among MET1, MET2, EcR and USP. Strong associations between bait and prey proteins led to more yeast colonies. (B) When the *HA-EcR*, *FLAG-USP*, and *V5-Met1* constructs were co-transfected into human HEK 293 cells, 20E treatment for 6 hr at a final concentration of 1 µM had little or no stimulating effects on the physical interactions between MET and EcR-USP. In the immunoprecipation experiments, the bottom Western blot is input. IP, immunoprecipitate; Blot, Western blot. (C) The *HA-EcR*, *FLAG-USP*, *V5-Met1*, and *cMyc-Met2* constructs were co-transfected into the human HEK 293 cells. After nuclear extracts were bound with biotin-labeled EcRE, the protein-DNA complexes were separated on a 5% native PAGE gel followed by EMSA. Addition of the HA or FLAG antibody resulted in a shift of EcRE. In (C) and (D), the shift was indicated by a black arrow in comparison with a gray arrow. (D) The *HA-EcR*, *FLAG-USP*, *V5-Met1*, and *cMyc-Met2* constructs were co-transfected into human HEK 293 cells. After nuclear extracts were bound with biotin-labeled EcRE, the protein-DNA complexes were separated 5% native PAGE followed by EMSA. When the V5 or cMyc antibody was added, binding of EcR-USP-EcRE was shifted by MET1 and MET2 in EMSA showing that MET, EcR-USP and EcRE form a protein-DNA complex. (E) *Met1* RNAi and transfection were simultaneously conducted in *Bombyx* DZNU-Bm-12 cells for 48 hr, followed by 20E treatment for 6 hr at a final concentration of 1 µM, and measurements of EcRE-driven luciferase activity were done. MET is required for 20E function to induce gene expression via the ecdysone receptor and EcRE. The bars labeled with different lowercase letters are significantly different (P<0.05, ANOVA).

It has been reported that *Drosophila* MET physically interacts with EcR-USP [36], [37]. A CytoTrap yeast two-hybrid experiment was carried out to investigate whether such direct associations among MET1, MET2, EcR and USP occur in *Bombyx*. As expected, EcR (the EcR-B1 isoform was used throughout the paper) and USP strongly associate with one another. Weak associations were observed between MET1 and MET2, MET1 and MET1, and MET2 and MET2, while intermediate associations were formed between the two MET proteins and the ecdysone receptor (Figure 5A). To confirm the yeast two-hybrid results that MET associates with EcR-USP, we performed immunoprecipitation experiments. When the *HA-EcR*, *FLAG-USP*, and *V5-Met1* constructs were co-transfected into human HEK 293 cells, MET physically interacts with EcR-USP, while 20E treatment had little or no stimulating effects on the physical interactions between MET1 and EcR-USP (Figure 5B) confirming the results reported in *Drosophila* [36], [37]. As negative controls, IgG was not able to pull down endogenous HA-EcR, FLAG-USP, or V5-MET1 (Figure S5A).

Since MET physically interacts with the ecdysone receptor, we investigated whether MET, ecdysone receptor and EcRE form a protein-DNA complex when the *HA-EcR*, *FLAG-USP*, *V5-Met1*, and *cMyc-Met2* constructs were co-transfected into HEK 293 cells. As expected, the overexpressed MET was not able to bind EcRE in the electrophoretic mobility shift assay (EMSA) but EcR-USP did (Figure S5B). Addition of the HA or FLAG antibody resulted in a shift of EcRE by HA-EcR or FLAG-USP (Figure 5C). When the V5 or cMyc antibody was added, binding of the ecdysone receptor-EcRE complex was also shifted by V5-MET1 or cMyc-MET2 (Figure 5D), demonstrating that MET, ecdysone receptor and EcRE form a protein-DNA complex.

Subsequently, *Met1* RNAi and *EcRE-Luc* transfections were simultaneously conducted in DZNU-Bm-12 cells, followed by 20E treatment and measurements of EcRE-driven luciferase activity. *Met1* RNAi had no apparent effects on basal luciferase activity, but significantly decreased 20E-induced EcRE-driven luciferase activity. Moreover, co-transfection with *EcR* and *USP* increased 20E-induced EcRE-driven luciferase activity, and this induction was again significantly decreased by *Met1* RNAi (Figure 5F), suggesting that MET is required for the maximal action of 20E in inducing gene expression via physical interaction with the ecdysone receptor and EcRE.

## Discussion

In this study, we demonstrate that MET is required for the maximal action of 20E in *Bombyx*. Although the *Met1* mRNA level in the fat body is much higher than the *Met2* mRNA level, with the first set of dsRNAs, the inhibiting effects of *Met2* RNAi on 20E-triggered gene expression and tissue remodeling were stronger than for *Met1* RNAi. However, results from the other two sets of dsRNAs showed that *Met1* RNAi had higher inhibiting effects than *Met2* RNAi. *Met* RNAi performed at different developmental stages and in cultured cells confirmed the major conclusion that MET is required for the maximal action of 20E in *Bombyx*. Unfortunately, it is difficult to determine with certainty which MET is more important and whether the two *Met* genes are functionally redundant in the 20E signal transduction pathway when only using RNAi methodology. One reason is that RNAi knockdown of one *Met* gene downregulates the other *Met* gene, which is 20E responsive. Our preliminary data suggest that the *Met1* mRNA level in the midgut was more abundant than the *Met2* mRNA level and that using the the first set of dsRNAs, *Met1* RNAi resulted in a more severe inhibitory effects on midgut remodeling than *Met2* RNAi. Since the systemic RNAi approach might result in non-tissue-autonomous effects, we suppose that performing RNAi with the binary GAL4/UAS system in *Bombyx* [38], [39] might be more useful for understanding which MET is more important in terms of different tissues or developmental stages. Mutation of the two *Met* genes, both separately and together, using a gene-targeting method [40] should be able to eventually resolve the problem in the future.

Both *Bombyx Met* genes are 20E responsive, exhibiting similar expression patterns to other 20E-response genes, including *Br-C*, *E74*, *E75* and *E93*. RNAi knockdown of each of these genes also interrupts the 20E-triggered transcriptional cascade to different levels (unpublished data). Thus, it appears that the 20E induction of those 20E-response genes (including the *Met* genes) is required for the maximal action of 20E to induce gene expression in *Bombyx*. In *Drosophila*, mutation of *Br-C*, *E74*, *E75*, or *E93* interrupts the 20E signaling, with more pronounced effects in *E93* mutants. It has been well documented that the 20E induction of E93 determines a PCD response by positively impacting the 20E signaling [19]. The feedback regulation of 20E signaling should be common in insects.

Previously, we demonstrated that MET and GCE are functionally redundant in transducing JH signal to induce *Kr-h1* expression and to antagonize 20E-induced *Br-C* expression [6], [7]. However, our preliminary experiments suggest that MET and GCE might be not the same in modulating 20E signaling using the *Met* and *gce* mutants. It might be incomparable between the function of *Met* and *gce* in *Drosophila* and that of *Met1* and *Met2* in *Bombyx*, since the duplication events in these two insect species are evolutionary independent [6], [41]. Nevertheless, in *Tribolium*, *Met* RNAi during the early quiescent stage also disrupts the 20E-triggered transcriptional cascade, showing that the single MET protein in *Tribolium* and the two MET proteins in *Bombyx* have similar functions in modulating 20E signaling during metamorphosis.

We have also tried to dissected out the molecular mechanism how MET is required for the maximal action of 20E in *Bombyx*. Our preliminary results suggest that MET might bind transcriptional co-activators, including CBP/p300 [42], which is important for transactivation of the 20E-ecdysone receptor complex via EcRE. The family of bHLH-PAS transcriptional regulators, consisting of transcription factors and co-activators, are critical components of gene expression networks that underlie essential developmental and environmental processes [43], [44]. The mammalian bHLH-PAS transcription factors, such as the dioxin receptor (DR), recruit many transcriptional co-activators, including CBP/p300, p160/SRC/NCoA, p140, and CARM1/PRMT1 for transactivation [45]. Ligand-activated nuclear receptors (i.e. estrogen receptor; ER) also recruit these transcriptional co-activators for transactivation [45]. Thus, it might be a common mechanism that a bHLH-PAS transcription factor recruits histone-modifying transcriptional co-activators to liganded nuclear receptors for transactivation [43], [45]. *CBP/p300* RNAi attenuated, but did not abolish, 20E-induced luciferase activity driven by EcRE, suggesting that the receptor complex may consist of other histone-modifying transcriptional co-activators. A good candidate is p160/SRC/NCoA, which has been demonstrated in *Aedes*, *Drosophila*, and *Tribolium* [20], [21], [22], [23].

Very recently, it has been documented that the *Bombyx* MET2 might act as a JH receptor. In the presence of JH, MET2 associates with SRC, the p160/SRC/NCoA-like molecule in *Bombyx*, to interact the JHRE in inducing *Kr-h1* expression [31]. Considering the MET function in both JH and 20E actions, we propose that MET plays a role mediating JH-20E crosstalk, and that the detailed molecular mechanism is surely worthy of further investigation.

## Materials and Methods

### Insects and cell lines

*Bombyx* (P50) [28], [29], *Tribolium* [9] were reared as previously described. *Bombyx* DZNU-Bm-12 cells [35] were maintained in TNM-FH (Sigma) medium supplemented with 10% heat-inactivated fetal bovine serum (Hyclone) at 27°C. And human HEK 293 cells were maintained in Dulbecco's Modified Eagle Medium (Hyclone) supplemented with 10% heat-inactivated fetal bovine serum (Hyclone).

### Conventional molecular, biochemical, and cellular approaches

The full-length *Met1* and *Met2* cDNA sequences (GenBank accession numbers: EU249371 and EU249372) were cloned using RACE. Details of qPCR and Western blotting were previously described [7], [27], [28], [46]. Caspase 3 activity was determined according to the manufacturer's instructions (Beyotime, Shanghai, China). TUNEL labeling (Beyotime) and LysoTracker staining (Invitrogen) were used to estimate apoptosis and autophagy, respectively, and monitored with an Olympus Fluoview FV1000 confocal microscope. Primers used here and elsewhere are listed in Table S1.

### RNAi and hormone treatment

dsRNAs were generated using the T7 RiboMAX™ Express RNAi System (Promega). Preliminary data showed that the P50 strain of *Bombyx* (the Chinese strain variation, Dazao) was more sensitive to RNAi treatments than the other tested strains [47], and the P50 strain of *Bombyx* was used throughout this study. RNAi knockdown was performed at two developmental stages, including initiation of the early wandering stage and 6 hr after pupation. After the RNAi treatment (10 or 30 µg of dsRNA per animal), fat body from the abdominal segments was collected for bioassays. Three biological replicates were used, each of which consisted of 10 silkworms. The details of hormone treatment *in vivo* (1 µg 20E per animal; Sigma Aldrich) were previously described [28], [29], [48].

RNAi knockdown in DZNU-Bm-12 cells was performed using the Effectene transfection reagent (Qiagen) for 48 hr at a final concentration of 2 µg/ml dsRNA. To determine the 20E primary-response genes, 1 µM 20E and 10 µg/ml cycloheximide (Sigma Aldrich) were used [48].

### Antibodies and immunohistochemistry

The *Bombyx* MET1 and Br-C antibodies were produced by the Abmart Company (Shanghai). A cDNA fragment encoding amino acids 151M to 350Q of MET1 and the full-length *Br-C* Z4 cDNA were expressed in *E. coli* and their protein products were purified. Antigen-purified rabbit polyclonal antibodies against MET1 and Br-C were generated. The AB11 USP-specific monoclonal antibody was provided by Dr. K.F. Kafatos (Harvard University). The monoclonal antibodies against the V5 tag (Sigma Aldrich), cMyc tag (Santa Cruz), and Tubulin (Invitrogen) were also used.

USP and Br-C were detected in explanted fat body from the 5^th^ abdominal segment by immunohistochemistry with the above primary antibodies. The fluorescein-conjugated secondary antibodies (Jackson ImmunoResearch) were FITC-conjugated Affinipure Goat Anti-Mouse IgG for USP and Cy3-conjugated Affinipure Goat Anti-Rabbit IgG for Br-C. Fluorescence signals were detected using the Olympus Fluoview FV1000 confocal microscope.

### Yeast two-hybrid assay

Yeast two-hybrid assays were carried out using the CytoTrap system (Stratagene), which is based on the ability of human Sos to complement a temperature-sensitive *cdc25* allele (*cdc25H*) in yeast when Sos is targeted to the plasma membrane through bait-prey interactions. This system has been well characterized for protein-protein interaction studies between transcription factors and their associated proteins (SR6). First, the full length *MET1*, *MET2*, *EcR-B1* (*EcR*) and *USP1* (*USP*) were amplified from silkworm genome, then these genes were cloned into bait or prey vector. MET1, MET2, EcR or USP was expressed as a fusion protein with human Sos as the bait protein. On the other hand, MET1, MET2, EcR and USP were expressed as prey proteins fused with a myristoylation (Myr) signal, targeting the proteins to the cell membrane. Expression of the prey is controlled by the GAL1 promoter, which is induced on galactose, and repressed on glucose medium. When bait and prey are co-transformed into the *cdc25H* strain, the only cells capable of growing at restrictive temperatures on galactose medium are those that have been rescued by the bait-prey interactions that recruit Sos to the cell membrane.

### Transient transfection assay

Transient transfection assay in DZNU-Bm-12 cells was carried out for 48 hr using Effectene according to the manufacturer's instructions. The final DNA concentration was 2 µg/ml, and the DNA∶Effectene ratio was 1∶25. The vector used to overexpress *V5-Met1* and *cMyc-Met2* was pEGFP-N1 (Clontech) under the control of the *ie1* promoter. After transfection, cells were treated with 1 µM 20E, followed by immunoprecipitation, qPCR, and luciferase assay.

Transient transfection assays in HEK 293 cells were performed using Lipofectamine 2000 (Invitrogen). The full length *MET1*, *MET2*, *EcR-B1* (*EcR*) and *USP* were amplified from the silkworm genome, and the *HA*, *FLAG*, *V5*, *cMyc* tags were fused in their 5-ends, respectively and cloned into the pcDNA 3.1(+) vector (Invitrogen). After transfection, the cells were harvested for immunopreciptation, luciferase assay and EMSA.

### Immunoprecipitation

After treatment, DZNU-Bm-12 cells and HEK 293 cells were harvested and lysed in ice-cold NP-40 lysis buffer (Beyotime). Lysates were incubated with FLAG, V5, or cMyc antibody or IgG for 4 hr, followed by incubation with protein G (GE Healthcare) overnight at 4°C. After extensive washing with cold NP-40 buffer, the samples were treated with RIPA lysis buffer (Beyotime) about 15min on the ice. Then immunoprecipitates were separated by SDS-PAGE and analyzed by Western blots after measured the protein concentration by the enhanced BCA protein assay kit (Beyotime).

### Luciferase assay

Luciferase assays were carried out using the Dual Luciferase Assay System (Promega) and a Modulus Luminometer (Turner BioSystems). The reporter pGL3 vector (Promega) containing four repeated EcRE sequences (GACAAGGGTTCAATGCACTTGTC) and a hsp70 mini promoter was used for the luciferase reporter. And the reference pRL vector (Promega) carrying Renila-luciferase driven by actin3 promoter was co-transfected into the cell with the reporter vector. The dual luciferase double reporter assay system and substrates were purchased from Promega.

### EMSA

The *HA-EcR*, *FLAG-USP*, *V5-Met1*, and *cMyc-Met2* constructs were co-transfected into HEK 293 cells and nuclear extracts were prepared by the NE-PER Nuclear and Cytoplasmic Extraction Reagents (Thermo). The minimal EcRE (sence: AGTTCAATGGCCT; anti-sense: AGGCCATTGAACT) was biotin-labeled as a probe using the Biotin 3′ End DNA Labeling Kit (Pierce). After binding, the nuclear extract (15 µg) containing the biotin-labeled EcRE and the protein-DNA complexes were separated on 5% nondenaturing PAGE gel. HA, FLAG, V5, and cMyc antibodies were added to the nuclear extract to detect the shift of EcRE. EMSA was performed using the LightShift Chemiluminescent EMSA Kit (Pierce).

## Supporting Information

Figure S1 **The diagram of the three sets of** ***Met*** **dsRNA and confirmation of the MET and Br-C antibodies.** (A) The diagram illustrates the three sets of *Met* dsRNA. Red bar: #1 set of *Met1* (491–916) and *Met2* (491–916) dsRNA; green bar: #2 set of *Met1* (141–586) and *Met2* (1925–2336) dsRNA; yellow bar: #3 set of *Met1* (948–1348) and *Met2* (245–669) dsRNA. (B and C) Western blotting confirmation of the MET1 and Br-C antibody after *Met1* and *Br-C* RNAi. The arrow points to the MET1 protein and the Br-C protein isoforms with ideal molecular weights. *efgp* RNAi was used as a control. Tubulin was used as a loading control.
(PDF)

Figure S2 ***Met*** **RNAi prevents removal of obsolete larval tissues and generation of adult structures.** dsRNA (10 µg per larva) was injected into larvae during initiation of the early wandering stage. More than 30 silkworms were used in each group. *egfp* dsRNA was used as a control. (A) *Met* RNAi larvae form thinner cocoons. Cocoon images were collected after the silkworms stopped spinning. (B) *Met* RNAi prevented silk gland lysis 24 hr after pupation. The inhibiting effects, particularly on the middle silk gland, by *Met2* RNAi were stronger than *Met1* RNAi. (C) *Met1* RNAi affected adult structure formation. Many of the surviving *Met1* RNAi treated adults exhibited shortened and distorted legs (left panel) or unexpanded wings (right panel).
(PDF)

Figure S3 ***Met*** **RNAi disrupts the 20E-triggered transcriptional cascade during the early wandering stage and in DZNU-Bm-12 Cells.** Three biological replicates were used and one was represented (A–C). In each biological replicate, more than 10 larvae were used (A and B). *egfp* dsRNA was used as a control. (A) The other two sets (#2 and #3) of *Met* dsRNA (30 µg per larva) also disrupt the 20E-triggered transcriptional cascade during initiation of the early wandering stage. See Figure S1A for the locations of the three sets of *Met* dsRNA. (B) 20E treatment fails to induce expression of 20E-response genes in fat body explanted from the *Met2* RNAi silkworms during the early wandering stage. (C) *Met1* RNAi disrupts the 20E-triggered transcriptional cascade, except *Met2* whose expression level is extremely low, in *Bombyx* DZNU-Bm-12 cells. RNAi knockdown was performed using the Effectene transfection reagent (Qiagen) for 48 hr at a final concentration of 2 µg/ml dsRNA. The cells were treated with 20E for 6 hr at a final concentration of 1 µM.
(PDF)

Figure S4 ***Met*** **is required for 20E action in** ***Tribolium*** **.** RNAi knockdown of either *EcR* or *Met* (∼4 ng per larva) in *Tribolium* during the early quiescent stage resulted in lethality (A), significantly delayed the larval-pupal transition (A and B), and disrupted the 20E-triggered transcriptional cascade (C). *egfp* dsRNA was used as a control. (A) Larval, prepupal, and pupal numbers were counted 24 and 48 hr after RNAi treatment. Total lethality caused by *egfp*, *Met* and *EcR* dsRNAs was compared. (B) Phenotypic images were collected from the above experimental animals 24 (left) and 48 hr (right) after RNAi treatment. (C) *Met*, *EcR*, *USP*, *E74*, and *Br-C* mRNA levels, as determined by qPCR, were significantly down-regulated 24 hr after *Met* RNAi.
(PDF)

Figure S5 **The negative controls for the IP and EMSA experiments.** (A) The *HA-EcR*, *FLAG-USP*, and *V5-Met1* constructs were co-transfected into human HEK 293 cells, the cells were treated by 20E for 6 hr at a final concentration of 1 µM. The negative control IgG was not able to pull down HA-EcR, FLAG-USP, and V5-Met1. IP, immunoprecipitate; Blot, Western blot. (B) The *HA-EcR* and *FLAG-USP* or *V5-Met1* and *cMyc-Met2* constructs were co-transfected into the human HEK 293 cells. After nuclear extracts were bound with biotin-labeled EcRE, the protein-DNA complexes were separated on a 5% native PAGE gel followed by EMSA. The shift was indicated by a black arrow in comparison with a gray arrow.
(PDF)

Table S1 **A list of all primers used in this paper.**
(DOC)
