# Supplementary figures and images for: MET Is Required for the Maximal Action of 20-Hydroxyecdysone during Bombyx Metamorphosis
Source: PLoS One. 2012 Dec 27;7(12):e53256. doi: 10.1371/journal.pone.0053256 (PMC3531340; doi:10.1371/journal.pone.0053256)

# Figure S1

**A**

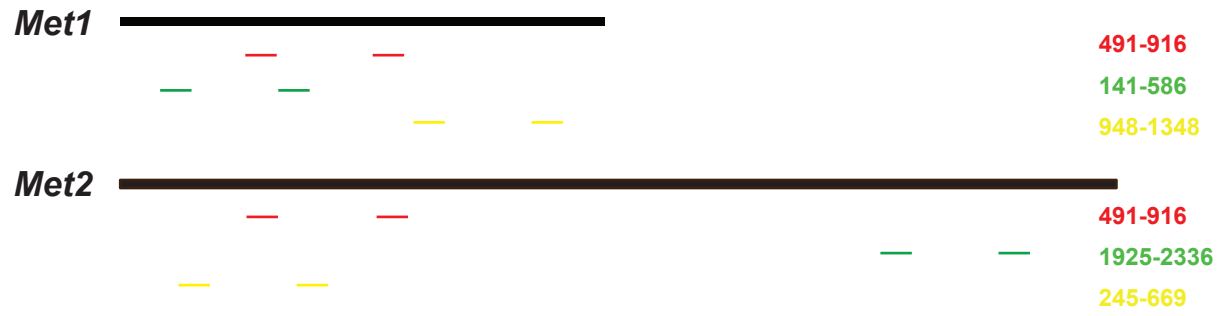

**B**

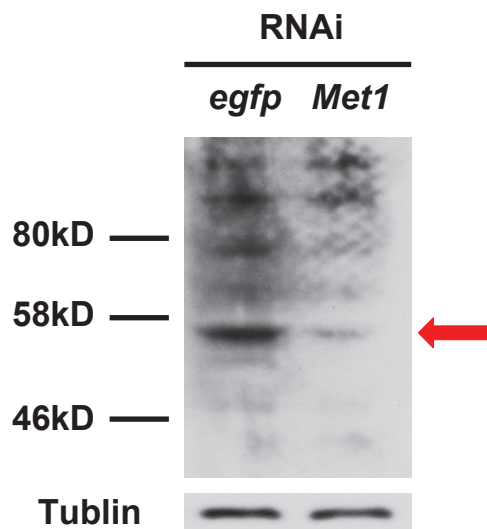

**C**

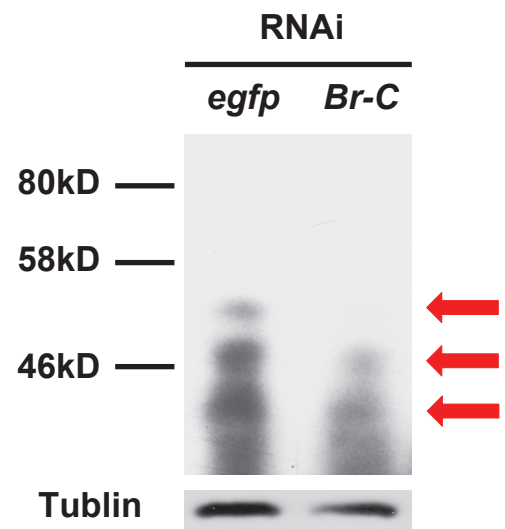

Supplement: Figure S1 — The diagram of the three sets of Met dsRNA and confirmation of the MET and Br-C antibodies. (A) The diagram illustrates the three sets of Met dsRNA. Red bar: #1 set of Met1 (491–916) and Met2 (491–916) dsRNA; green bar: #2 set of Met1 (141–586) and Met2 (1925–2336) dsRNA; yellow bar: #3 set of Met1 (948–1348) and Met2 (245–669) dsRNA. (B and C) Western blotting confirmation of the MET1 and Br-C antibody after Met1 and Br-C RNAi. The arrow points to the MET1 protein and the Br-C protein isoforms with ideal molecular weights. efgp RNAi was used as a control. Tubulin was used as a loading control. (PDF) [file pone.0053256.s001.pdf]

Figure S2

A

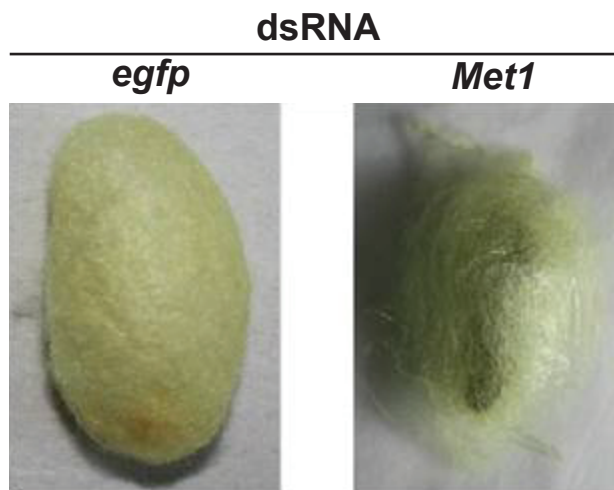

B

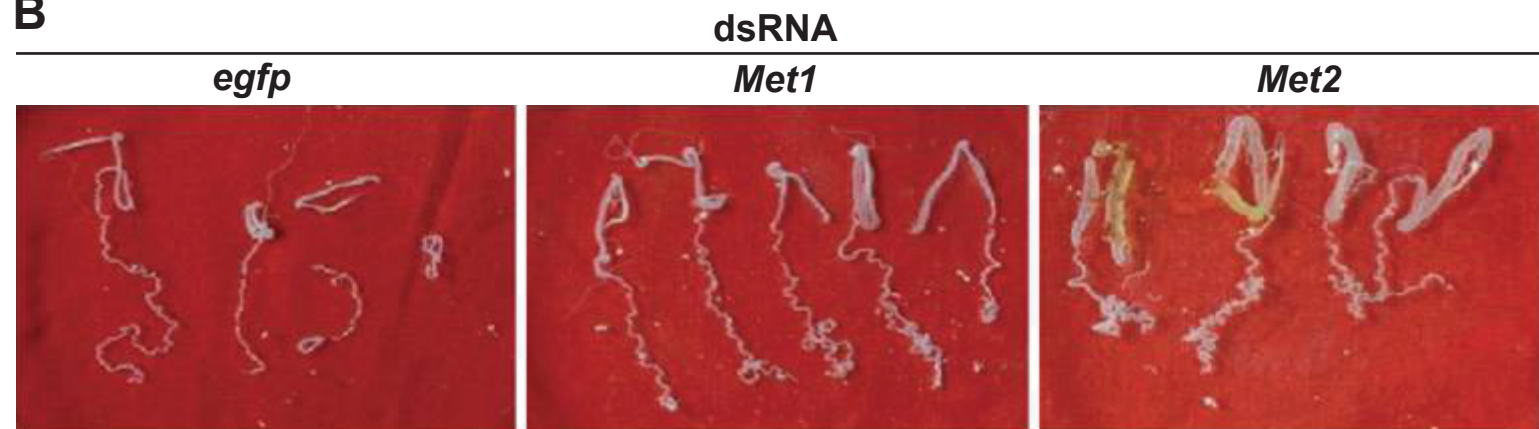

C

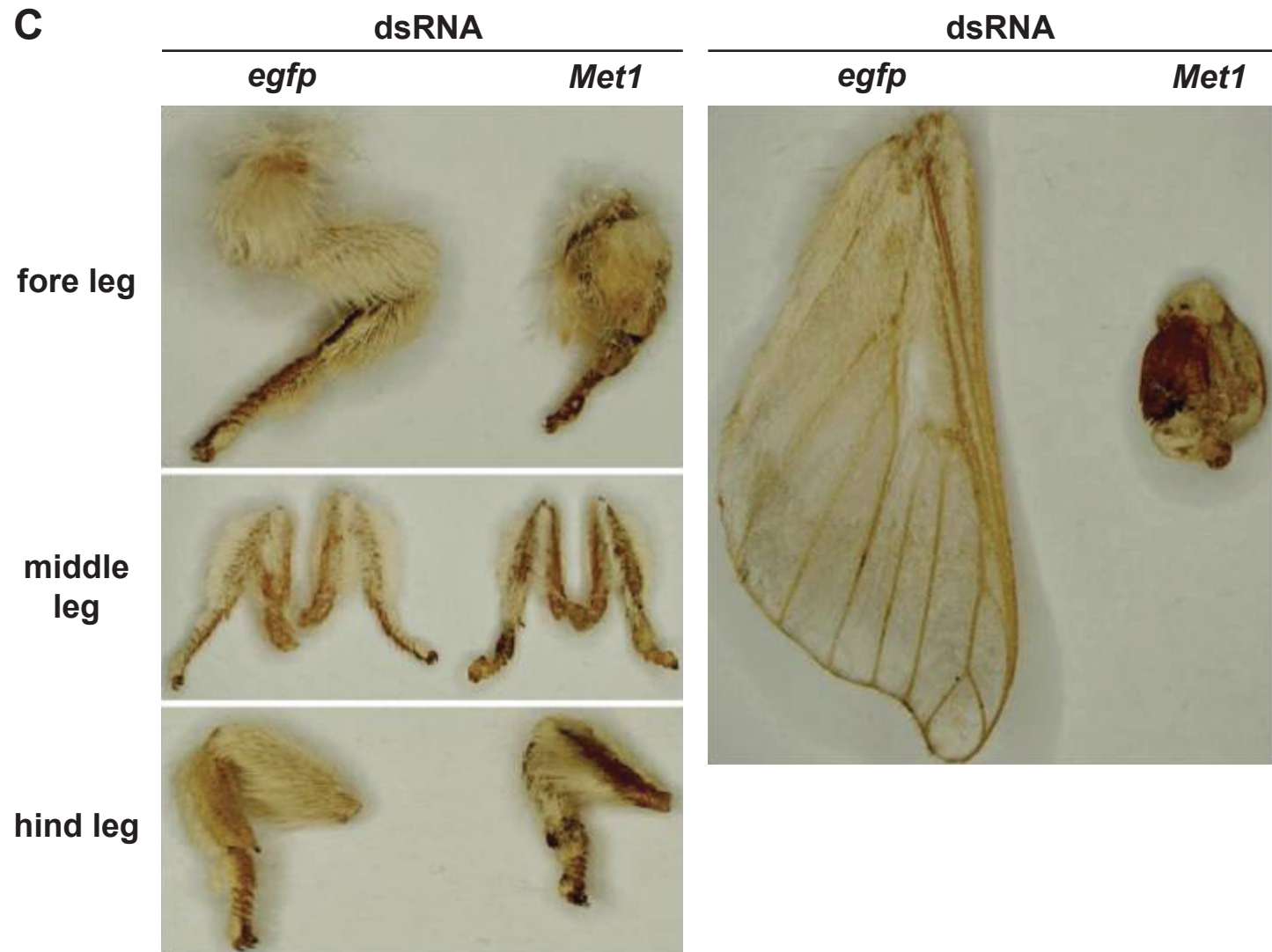

Supplement: Figure S2 — Met RNAi prevents removal of obsolete larval tissues and generation of adult structures. dsRNA (10 µg per larva) was injected into larvae during initiation of the early wandering stage. More than 30 silkworms were used in each group. egfp dsRNA was used as a control. (A) Met RNAi larvae form thinner cocoons. Cocoon images were collected after the silkworms stopped spinning. (B) Met RNAi prevented silk gland lysis 24 hr after pupation. The inhibiting effects, particularly on the middle silk gland, by Met2 RNAi were stronger than Met1 RNAi. (C) Met1 RNAi affected adult structure formation. Many of the surviving Met1 RNAi treated adults exhibited shortened and distorted legs (left panel) or unexpanded wings (right panel). (PDF) [file pone.0053256.s002.pdf]

# Figure S3

**A**

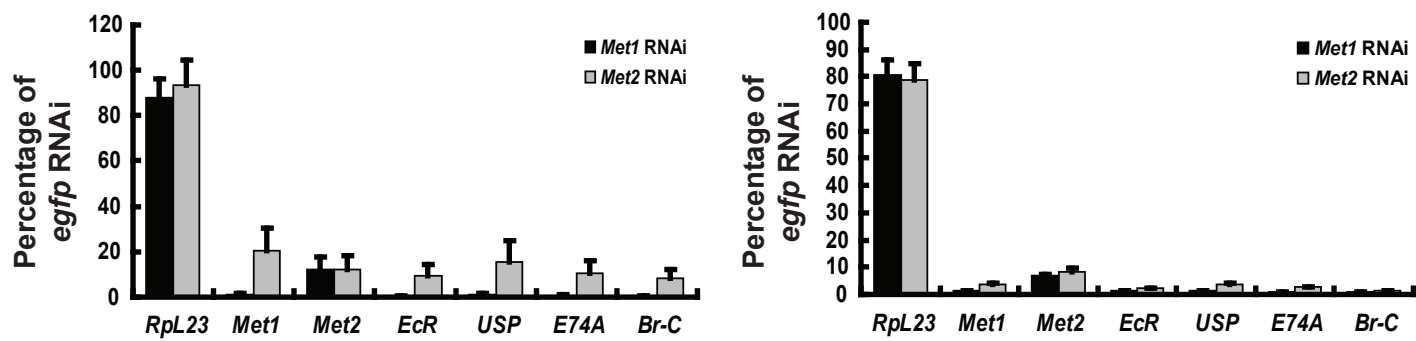

**B**

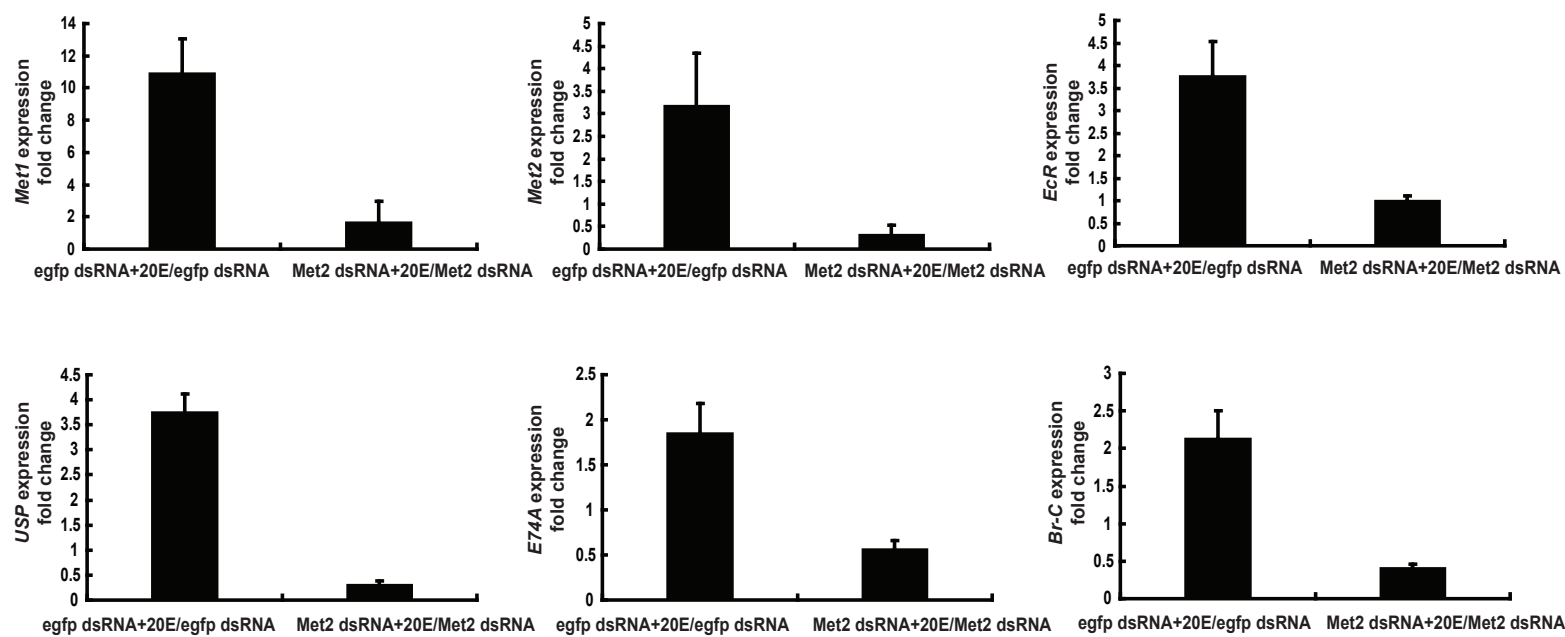

**C**

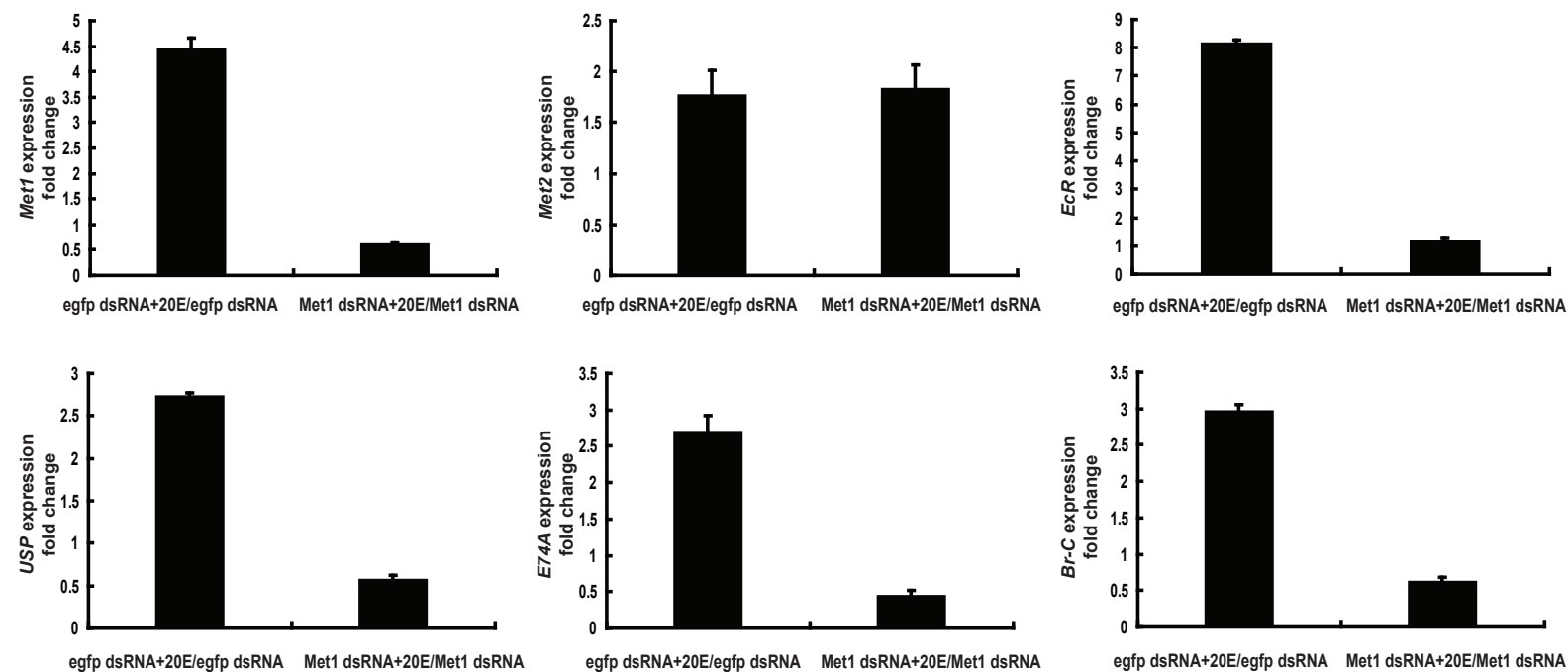

Supplement: Figure S3 — Met RNAi disrupts the 20E-triggered transcriptional cascade during the early wandering stage and in DZNU-Bm-12 Cells. Three biological replicates were used and one was represented (A–C). In each biological replicate, more than 10 larvae were used (A and B). egfp dsRNA was used as a control. (A) The other two sets (#2 and #3) of Met dsRNA (30 µg per larva) also disrupt the 20E-triggered transcriptional cascade during initiation of the early wandering stage. See Figure S1A for the locations of the three sets of Met dsRNA. (B) 20E treatment fails to induce expression of 20E-response genes in fat body explanted from the Met2 RNAi silkworms during the early wandering stage. (C) Met1 RNAi disrupts the 20E-triggered transcriptional cascade, except Met2 whose expression level is extremely low, in Bombyx DZNU-Bm-12 cells. RNAi knockdown was performed using the Effectene transfection reagent (Qiagen) for 48 hr at a final concentration of 2 µg/ml dsRNA. The cells were treated with 20E for 6 hr at a final concentration of 1 µM. (PDF) [file pone.0053256.s003.pdf]

Figure S5

A

| IP  | Blot     | 20E |   |
|-----|----------|-----|---|
|     |          | -   | + |
| IgG | HA-EcR   |     |   |
|     | FLAG-USP |     |   |
|     | V5-MET1  |     |   |

B

|                  |   |   |
|------------------|---|---|
| <i>HA-EcR</i>    | - | + |
| <i>FLAG-USP</i>  | - | + |
| <i>V5-MET1</i>   | + | - |
| <i>cMyc-MET2</i> | + | - |

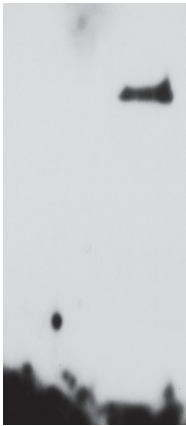

Supplement: Figure S5 — The negative controls for the IP and EMSA experiments. (A) The HA-EcR, FLAG-USP, and V5-Met1 constructs were co-transfected into human HEK 293 cells, the cells were treated by 20E for 6 hr at a final concentration of 1 µM. The negative control IgG was not able to pull down HA-EcR, FLAG-USP, and V5-Met1. IP, immunoprecipitate; Blot, Western blot. (B) The HA-EcR and FLAG-USP or V5-Met1 and cMyc-Met2 constructs were co-transfected into the human HEK 293 cells. After nuclear extracts were bound with biotin-labeled EcRE, the protein-DNA complexes were separated on a 5% native PAGE gel followed by EMSA. The shift was indicated by a black arrow in comparison with a gray arrow. (PDF) [file pone.0053256.s005.pdf]
