# Supplementary material for: MET Is Required for the Maximal Action of 20-Hydroxyecdysone during Bombyx Metamorphosis
Source: PLoS One. 2012 Dec 27;7(12):e53256. doi: 10.1371/journal.pone.0053256 (PMC3531340; doi:10.1371/journal.pone.0053256)

Figure S4

A

dsRNA	Injected larvae	Larvae,%		Prepupae,%		Pupae,%		Total lethality,%
		24h	48h	24h	48h	24h	48h	
egfp	19	31.6	0	0	5.3	68.4	94.7	5.3
Met	33	69.7	60.7	24.2	30.3	6.1	9	81.8
EcR	22	90.9	77.3	9.1	22.7	0	0	90.9

B

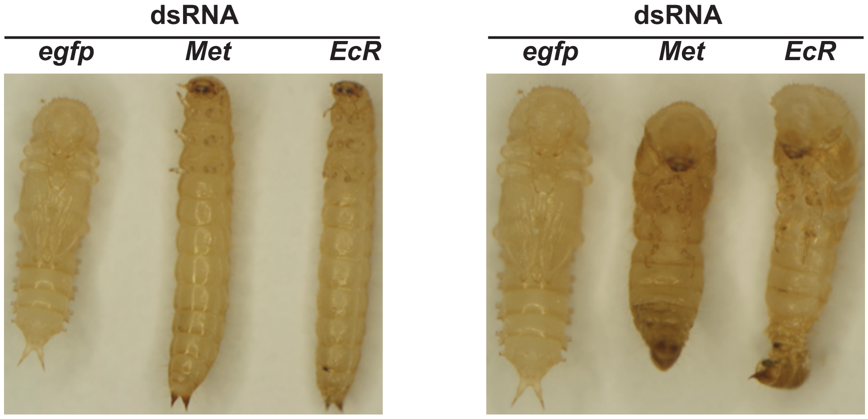

C

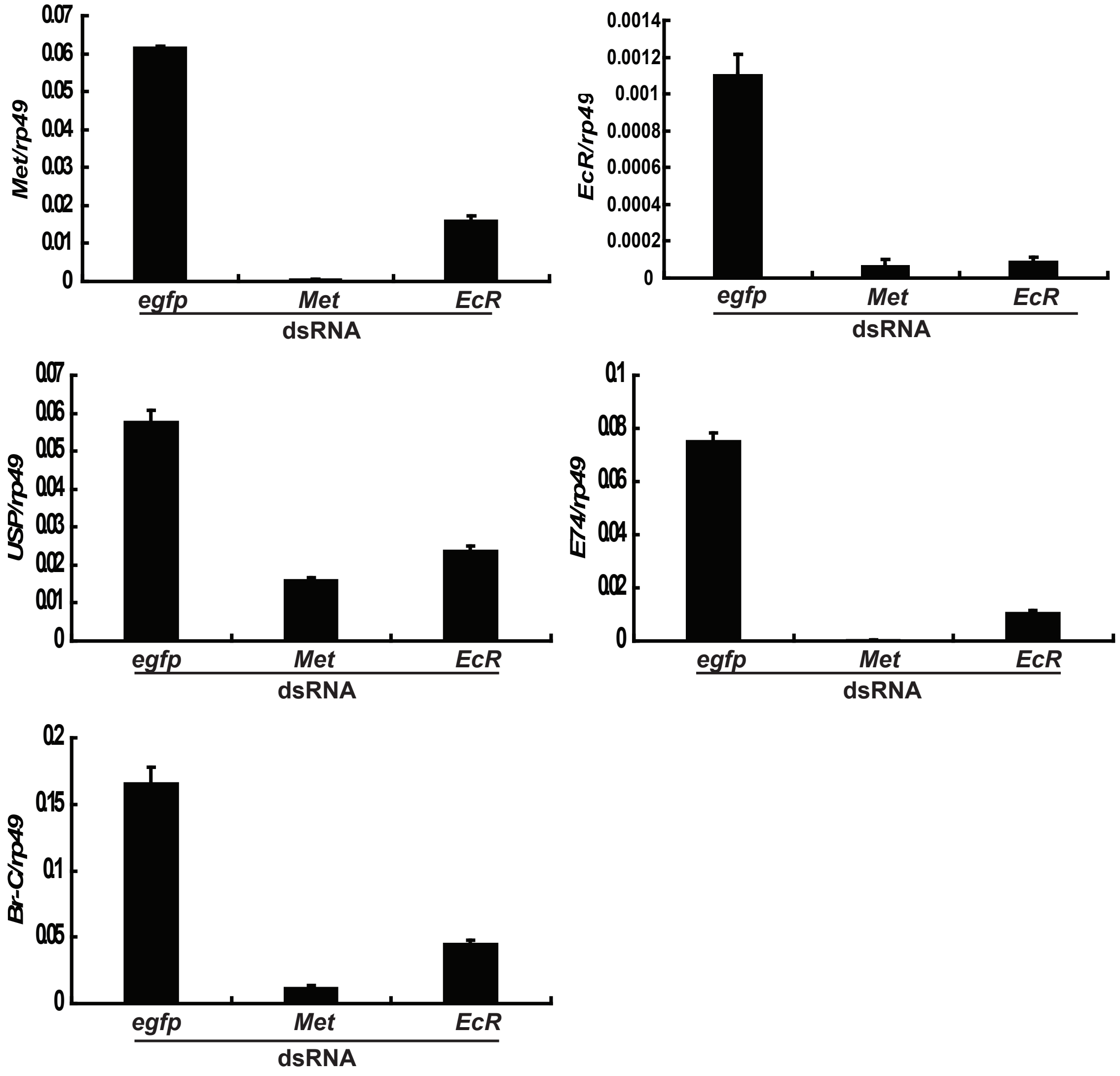

Supplement: Figure S4 — Met is required for 20E action in Tribolium . RNAi knockdown of either EcR or Met (∼4 ng per larva) in Tribolium during the early quiescent stage resulted in lethality (A), significantly delayed the larval-pupal transition (A and B), and disrupted the 20E-triggered transcriptional cascade (C). egfp dsRNA was used as a control. (A) Larval, prepupal, and pupal numbers were counted 24 and 48 hr after RNAi treatment. Total lethality caused by egfp, Met and EcR dsRNAs was compared. (B) Phenotypic images were collected from the above experimental animals 24 (left) and 48 hr (right) after RNAi treatment. (C) Met, EcR, USP, E74, and Br-C mRNA levels, as determined by qPCR, were significantly down-regulated 24 hr after Met RNAi. (PDF) [file pone.0053256.s004.pdf]
