# Supplementary material for: MET Is Required for the Maximal Action of 20-Hydroxyecdysone during Bombyx Metamorphosis
Source: PLoS One. 2012 Dec 27;7(12):e53256. doi: 10.1371/journal.pone.0053256 (PMC3531340; doi:10.1371/journal.pone.0053256)
Supplement: Table S1 — A list of all primers used in this paper. (DOC) [file pone.0053256.s006.doc]

**Supporting information:**

**Figure S1 The diagram of the three sets of *Met* dsRNA and confirmation of the MET and Br-C antibodies.**

(A) The diagram illustrates the three sets of *Met* dsRNA. Red bar: #1 set of *Met1* (491-916) and *Met2* (491-916) dsRNA; green bar: #2 set of *Met1* (141-586) and *Met2* (1925-2336) dsRNA; yellow bar: #3 set of *Met1* (948-1348) and *Met2* (245-669) dsRNA.

(B and C) Western blotting confirmation of the MET1 and Br-C antibody after *Met1* and *Br-C* RNAi. The arrow points to the MET1 protein and the Br-C protein isoforms with ideal molecular weights. *efgp* RNAi was used as a control. Tubulin was used as a loading control.

**Figure S2 *Met* RNAi prevents removal of obsolete larval tissues and generation of adult structures.**

dsRNA (10 µg per larva) was injected into larvae during initiation of the early wandering stage. More than 30 silkworms were used in each group. *egfp* dsRNA was used as a control.

1. *Met* RNAi larvae form thinner cocoons. Cocoon images were collected after the silkworms stopped spinning.
2. *Met* RNAi prevented silk gland lysis 24 hr after pupation. The inhibiting effects, particularly on the middle silk gland, by *Met2* RNAi were stronger than *Met1* RNAi.
3. *Met1* RNAi affected adult structure formation. Many of the surviving *Met1* RNAi treated adults exhibited shortened and distorted legs (left panel) or unexpanded wings (right panel).

**Figure S3 *Met* RNAi disrupts the 20E-triggered transcriptional cascade during the early wandering stage and in DZNU-Bm-12 Cells.**

Three biological replicates were used and one was represented (A-C). In each biological replicate, more than 10 larvae were used (A and B). *egfp* dsRNA was used as a control.

1. The other two sets (#2 and #3) of *Met* dsRNA (30 µg per larva) also disrupt the 20E-triggered transcriptional cascade during initiation of the early wandering stage. See Figure S1A for the locations of the three sets of *Met* dsRNA.
2. 20E treatment fails to induce expression of 20E-response genes in fat body explanted from the *Met2* RNAi silkworms during the early wandering stage.
3. *Met1* RNAi disrupts the 20E-triggered transcriptional cascade, except *Met2* whose expression level is extremely low, in *Bombyx* DZNU-Bm-12 cells. RNAi knockdown was performed using the Effectene transfection reagent (Qiagen) for 48 hr at a final concentration of 2 µg/ml dsRNA. The cells were treated with 20E for 6 hr at a final concentration of 1 μM.

**Figure S4 *Met* is required for 20E action in *Tribolium*.**

RNAi knockdown of either *EcR* or *Met* (~4 ng per larva) in *Tribolium* during the early quiescent stage resulted in lethality (A), significantly delayed the larval-pupal transition (A and B), and disrupted the 20E-triggered transcriptional cascade (C). *egfp* dsRNA was used as a control.

1. Larval, prepupal, and pupal numbers were counted 24 and 48 hr after RNAi treatment. Total lethality caused by *egfp*, *Met* and *EcR* dsRNAs was compared.
2. Phenotypic images were collected from the above experimental animals 24 (left) and 48 hr (right) after RNAi treatment.
3. *Met*, *EcR*, *USP*, *E74*, and *Br-C* mRNA levels, as determined by qPCR, were significantly down-regulated 24 hr after *Met* RNAi.

**Figure S5 The negative controls for the IP and EMSA experiments.**

1. The *HA-EcR*, *FLAG-USP*, and *V5-Met1* constructs were co-transfected into human HEK 293 cells, the cells were treated by 20E for 6 hr at a final concentration of 1 μM. The negative control IgG was not able to pull down HA-EcR, FLAG-USP, and V5-Met1. IP, immunoprecipitate; Blot, Western blot.
2. The *HA-EcR* and *FLAG-USP* or *V5-Met1* and *cMyc-Met2* constructs were co-transfected into the human HEK 293 cells. After nuclear extracts were bound with biotin-labeled EcRE, the protein-DNA complexes were separated on a 5% native PAGE gel followed by EMSA. The shift was indicated by a black arrow in comparison with a gray arrow.

**Table S1. A list of all primers used in this paper**

*The bold sequence means the minimal T7 RNA polymerase promoter sequence.

| **primer** | **sequence** |
| --- | --- |

| *egfp* RNAi template sense primer | **TAATACGACTCACTATAGG**GAGAATGGTGAGCAAGGGCG |
| --- | --- |
| *egfp* RNAi template antisense primer | **TAATACGACTCACTATAGG**GAGACTTGTACAGCTCGTCC |
| *BmMet1 #1* RNAi template sense primer | **GGATCCTAATACGACTCACTATAGG**AATTAGGTTATTGCCATATAGATTTAGTA |
| *BmMet1 #1* RNAi template antisense primer | **GGATCCTAATACGACTCACTATAGG**CTATCAAATGTCTCGTCCAATATTC |
| *BmMet2* #1 RNAi template sense primer | **GGATCCTAATACGACTCACTATAGG**TGAAATTATTCAACGGATTTTTGAT |
| *BmMet2* #1 RNAi template antisense primer | **GGATCCTAATACGACTCACTATAGG**CTTCGCCACGTGTACGAGAG |
| *BmMet1 #2* RNAi template sense primer | **GGATCCTAATACGACTCACTATAGG**TTCTTCAGATTCACGTTCGC |
| *BmMet1 #2* RNAi template antisense primer | **GGATCCTAATACGACTCACTATAGG**GGTGGGTAAATGTGACAACG |
| *BmMet2* #2 RNAi template sense primer | **GGATCCTAATACGACTCACTATAGG**TGACGGCTACTTCACCTGAC |
| *BmMet2* #2 RNAi template antisense primer | **GGATCCTAATACGACTCACTATAGG**GATGGTTTGCTCCAAGGATT |
| *BmMet1 #3* RNAi template sense primer | **GGATCCTAATACGACTCACTATAGG**ATCATTGGCAGCTGGTTACA |
| *BmMet1 #3* RNAi template antisense primer | **GGATCCTAATACGACTCACTATAGG**GCATTACAATCCGCTCAAGA |
| *BmMet2* #3 RNAi template sense primer | **GGATCCTAATACGACTCACTATAGG**CACCCAAAGTGGAGAGTCCT |
| *BmMet2* #3 RNAi template antisense primer | **GGATCCTAATACGACTCACTATAGG**GCTTCTTGGCTTCAGCTTCT |
| *Bmrp49* qRT-PCR sense primer | CAGGCGGTTCAAGGGTCAATAC |
| *Bmrp49* qRT-PCR antisense primer | TGCTGGGCTCTTTCCACGA |
| *BmMet1* qRT-PCR sense primer | TCACGTTCGCCAGAGACTAC |
| *BmMet1* qRT-PCR antisense primer | GTCAACTTTCCGTTGGGAAT |
| *BmMet2* qRT-PCR sense primer | AAACGGCCATTAAATCCTTG |
| *BmMet2* qRT-PCR antisense primer | TGAACCTCGGATGAAATCAA |
| *BmEcR* qRT-PCR sense primer | GCTGGTCTGATAACGGTGGCT |
| *BmEcR* qRT-PCR antisense primer | CAAGGATTCCGGCGACATAAC |
| *BmUSP* qRT-PCR sense primer | AATGTCGGTAACTGCGTTGA |
| *BmUSP* qRT-PCR antisense primer | TCGAGTTCAACATTGGGTGT |
| *BmE74A* qRT-PCR sense primer | AGCAGTCAACTGCAAGGGTA |
| *BmE74A* qRT-PCR antisense primer | GTGCCCGATCTAAGGAGTTG |
| *BmBr-C* qRT-PCR sense primer | AAGACGTGGCGTACACAGAC |
| *BmBr-C* qRT-PCR antisense primer | TCAGGAATGAGGACAAGCTG |
| *BmRpL23* qRT-PCR sense primer | AAGAGAGGACGTGGTGGTTC |
| *BmRpL23* qRT-PCR antisense primer | AGTCTGTTCAGGCGACCTTT |
| EcRE sense primer | AGTTCAATGGCCT |
| EcRE antisense primer | AGGCCATTGAACT |
| *TcMet* RNAi template sense primer | **GGATCCTAATACGACTCACTATAGG**TAAGGCGGCAAACTC |
| *TcMet* RNAi template antisense primer | **GGATCCTAATACGACTCACTATAGG**TGGCTCAACCGACTCGTC |
| *TcEcR* RNAi template sense primer | **GGATCCTAATACGACTCACTATAGG**AGGCATATCACGGAAATTACCAT |
| *TcEcR* RNAi template antisense primer | **GGATCCTAATACGACTCACTATAGG**ATCTCGGAATTTTGGTTGCCT |
| *Tcrp49* qRT-PCR sense primer | TTATGGCAAACTCAAACGCAAC |
| *Tcrp49* qRT-PCR antisense primer | GGTAGCATGTGCTTCGTTTTG |
| *TcMet* qRT-PCR sense primer | GGGAAAGCAAAGGATCATCA |
| *TcMet* qRT-PCR antisense primer | AAGGCCTTCTTGCTCACTCA |
| *TcEcR* qRT-PCR sense primer | TTACCACTACAACGCCCTCA |
| *TcEcR* qRT-PCR antisense primer | TCTATCTCGCAATTGTTGCC |
| *TcUSP* qRT-PCR sense primer | GAGAAACGAGTCGAATGCAA |
| *TcUSP* qRT-PCR antisense primer | GGCAATGATGTGAAATGAGG |
| *TcBr-C* qRT-PCR sense primer | TGAACGAGGAGAACGTGAAG |
| *TcBr-C* qRT-PCR antisense primer | GATTATTAGGCCCGTTGTCG |
| *TcE74* qRT-PCR sense primer | AAGACACCCATTGTCATGGA |
| *TcE74* qRT-PCR antisense primer | AATCCGGTATGGAGGTGAAG |
